# Risk factors and surgical outcomes in pediatric patients with congenital heart disease and ischemic colitis

**DOI:** 10.1007/s00383-024-05950-3

**Published:** 2024-12-26

**Authors:** N. Mokhaberi, E. P. Schneider, M. Aftzoglou, I. Hüners, M. Körner, L. Armbrust, D. Biermann, R. Kozlik-Feldmann, M. Hübler, K. Reinshagen, C. Tomuschat

**Affiliations:** 1 Department of Pediatric Surgery, University Medical Center Hamburg-Eppendorf, Hamburg, Germany; 2 University Heart and Vascular Center Hamburg, University Medical Center Hamburg-Eppendorf, Hamburg, Germany; 3 Department of Paediatric Surgery, Norfolk and Norwich University Hospital, Norwich, UK; 4 Department of Congenital and Pediatric Heart Surgery, University Heart & Vascular Center, University Medical Center Hamburg-Eppendorf, Hamburg, Germany; 5 German Center for Child and Adolescent Health (DZKJ), Partner Site Hamburg, University Medical Center Hamburg-Eppendorf, Hamburg, Germany

**Keywords:** Ischemic colitis, Congenital heart defect, Bowel ischemia, Pediatric, Necrotizing enterocolitis

## Abstract

**Introduction:**

This study investigates risk factors and surgical outcomes in pediatric patients with congenital heart defects (CHD) who develop ischemic colitis (IC). Previous research indicates a higher IC risk in very low birth weight neonates with CHD.

**Methods:**

A retrospective analysis compared an IC-CHD group to a CHD-only group. Key variables included gestational age, birth weight, multiple pregnancies, prematurity, APGAR scores, cardiac and surgical characteristics, Aristotle-Score, and mortality rates. Surgical outcomes such as bowel resection and stoma procedures were also evaluated.

**Results:**

IC-CHD exhibited significantly lower gestational ages and birth weights, with higher rates of multiple pregnancies and prematurity. APGAR scores were notably lower. Cardiac and surgical data showed more frequent ECMO use and shorter cardiopulmonary bypass durations in the IC-CHD group. High rates of bowel resection highlighted severe gastrointestinal involvement. Mortality was significantly higher in IC-CHD with elevated Aristotle scores correlating with poorer outcomes.

**Conclusion:**

Gestational age, birth weight, and initial health status are critical in predicting IC risk and surgical outcomes in pediatric patients with CHD. The significantly higher mortality and complex surgical needs in the IC-CHD group underscore the necessity for vigilant monitoring and tailored interventions. Development of targeted therapeutic strategies adjustment for confounding factors in future studies is needed.

## Introduction

Ischemic colitis (IC) is caused by hypoperfusion to the colon; eventually leading to hemorrhage of the intestinal mucosa [1]. In pediatric patients, it is often linked to congenital heart disease (CHD) with altered intestinal perfusion [2]. In contrast to IC, necrotizing enterocolitis (NEC) is a common condition in premature infants characterized by intestinal inflammation, hypoxia and eventual intestinal necrosis resulting from intestinal immaturity, abnormal microbial colonization and inadequate circulatory regulation [3–6]. It should be noted that the term “necrotizing enterocolitis” (NEC) includes various NEC-like conditions of patients with intestinal injury and is often used synonymously. In term and late preterm infants the diagnosis NEC is often used in connection with conditions which lead to impaired bowel perfusion like CHD or perinatal hypoxia; thus ischemic colitis (IC) [3].

Similarly to NEC and NEC-like lesions like IC, CHD, the most prevalent single-organ congenital anomaly, shares this burden of infant morbidity and mortality. Neonates with CHD face the risk of developing IC. Simple CHDs remain stable in incidence since 1996, while complex CHDs have seen a significant rise since 2008 [7]. In these CHD patients, compromised cardiac function leads to insufficiently oxygenated blood and reduced bowel wall blood flow, increasing the risk of bowel resection. Certain types of CHD, such as patent ductus arteriosus, significant left-to-right shunting, and atrioventricular canal defect, have been linked to IC development, and even after surgery, ischemia and reperfusion injury remain potential threats [8–10]. Notably, hypoplastic left heart syndrome, truncus arteriosus, and aortopulmonary window carry the highest IC risk, particularly in premature or very low birth weight infants. Atrioventricular canal defects are a significant risk factor in this population [11, 12]. Other possible risk factors include high doses of prostaglandin, poor systemic perfusion, the use of cardiopulmonary bypass or red blood cell transfusion [11, 13, 14].

A review of the current literature shows many studies comparing cardiogenic NEC/IC with classic NEC in premature infants. In contrast, our objective was to identify potential risk factors for the onset of IC in a cohort of patients with CHD. In addressing this issue, it is essential to consider not only the surgical aspects of cardiac surgery or the cardiac defect itself, but also the entirety of the preoperative, perioperative, and postoperative management.

Therefore, our study aimed to identify CHD patients who underwent surgery for suspected IC and evaluate the associated risk factors, enabling early detection and optimized care.

## Methods

### Data collection and inclusion criteria

This retrospective study encompassed all patients with congenital heart defects (CHD) who underwent surgery for IC before or after cardiac surgery or intervention at the Department of Pediatric Surgery, University Medical Center Hamburg-Eppendorf (UKE) from January 2013 to May 2023 (IC-CHD group). The patients were identified using the International Statistical Classification of Diseases and Related Health Problems (ICD) codes. In view of the aforementioned overlaps, codes for both IC and NEC were included. Subsequently, the cases were differentiated on the basis of the presence of cardiac diagnoses and the surgical report. Controls were all patients with CHD up to the age of 18 months who underwent cardiac surgery or intervention without episodes of surgically treated IC in 2023 (CHD group). The study adhered to the principles outlined in the Helsinki Declaration and received approval from the Hamburg Ethics Committee (2023–101012-BO-ff).

Demographic and surgical data collected included gestational age (GA), gender, multiple gestation, gestational weight (GW), prematurity, syndromic condition, APGAR scores, presence of a cyanotic heart defect, complexity of CHD, extracorporeal membrane oxygenation (ECMO) use, duration of cardiopulmonary bypass (CPB), temporal relationship between heart and intestinal surgery, bowel resection, stoma creation, Aristotle-Score, type of patent ductus arteriosus (PDA) closure, and instances of mortality.

The primary endpoint was to identify risk factors for developing ischemic colitis in pediatric patients with CHD. Secondary endpoints included the temporal and surgical characteristics of bowel surgery and the relationship between surgical procedures and cardiac intervention.

### Aristotle-score

The Aristotle-Score was founded in 1999 by four major international societies of pediatric cardiac surgery to evaluate the quality of care in congenital heart surgery based on the complexity of operations. It can be divided into the Basic Score, which evaluates the complexity based on the potential mortality, morbidity and technical difficulty, and the Comprehensive Score, which includes the specific patient characteristics [15, 16].

### Statistical analysis

Statistical analyses were performed using R language (R Core Team: R: A Language and Environment for Statistical Computing, Vienna, Austria: Foundation for Statistical Computing). Continuous variables were expressed as mean ± standard deviation and as median with interquartile range (IQR). Categorical variables were expressed as frequencies (n) and percentages (%). Categorical data were analyzed using the Chi-square test or Fisher’s exact test. Continuous variables were checked for deviation from normal distribution using the Kolmogorov–Smirnoff normality test. Student’s t-Test or Mann–Whitney U Test were performed for continuous data as appropriate. For all analyses, a 5% significance level was set, and p-values were two-tailed.

## Results

The study included 37 patients in the IC-CHD group and 116 patients in the CHD group. Key demographic and baseline characteristics, cardiac and surgical characteristics, and surgical outcomes are presented below (Tables 1, 2, 3, 4, 5, 6).

**Table 1:** Demographic and Clinical Characteristics

Characteristic	CHD (N = 116)	IC-CHD (N = 37)	P-value
Multiple pregnancy (%)	4.3	24.3	< 0.001 (C)
Gestational age (weeks, mean ± SD)	37.6 ± 3.95	33.6 ± 5.71	< 0.001 (M-W)
Prematurity (%)	15.5	56.8	< 0.001 (C)
Syndromic condition (%)	14.7	27.0	0.141 (C)
Gestational weight (grams, mean ± SD)	3080 ± 867	2040 ± 1200	< 0.001 (M-W)
Apgar Minute 1 (mean ± SD)	7.39 ± 2.03	6.22 ± 2.52	0.015 (M-W)
Apgar Minute 5 (mean ± SD)	8.53 ± 1.76	7.97 ± 1.36	0.014 (M-W)
Apgar Minute 10 (mean ± SD)	9.18 ± 1.02	8.75 ± 0.98	0.013 (M-W)

**Table 2:** Cardiac and Surgical Characteristics

Characteristic	CHD (N = 116)	IC-CHD (N = 37)	P-value
Cyanotic heart defect (%)	47.4	37.8	0.269 (C)
Complexity of heart defect (%)			0.714 (C)
Mild	25.9	29.7	
Moderate	48.3	40.5	
Severe	25.9	29.7	
ECMO use (%)	3.4	10.8	0.097 (F)
ECMO duration (days, mean ± SD)	4.50 ± 0.71	19.5 ± 27.5	1.000 (M-W)
CPB duration (minutes, mean ± SD)	87.1 ± 61.1	40.8 ± 78.3	< 0.001 (M-W)

**Table 3:** Cardiac Diagnoses in IC-CHD Group

	N
PDA	9
Ventricular Septal Defect (VSD)	4
Pulmonary Atresia (PA)	3
d-Transposition of the Great Arteries (d-TGA)	2
Dilated Cardiomyopathy (DCM)	2
Pulmonary Stenosis (PS)	2
Tetralogy of Fallot (TOF)	2
Double Outlet Right Ventricle (DORV)	2
Borderline Left Ventricle	1
PA + VSD	1
Hypoplastc Left Heart Syndrom (HLHS)	1
Supraventricular Tachycardia (SVT)	1
Valvular Aortic Stenosis (AS, v)	1
Interrupted Aortic Arch Type B (IAA)	1
Complete Atrioventricular Septal Defect (cAVSD)	1
Atrial Septal Defect (ASD)	1
Coarctation of the Aorta (COA)	1
Common Arterial Trunc (TAC)	1
Pulmonary vein stenosis	1

**Table 4:** Surgical Outcomes in IC-CHD Group

Characteristic	IC-CHD (N = 37)
IC prior/after cardiac intervention (%)	
After	62.2
No cardiac surgery	27.0
Prior	10.8
Age at time of bowel surgery (days, mean ± SD)	222 ± 630
Time between cardiac and bowel surgery (days, mean ± SD)	13.2 ± 33.5
Time between start of IC and bowel surgery (days, mean ± SD)	1.19 ± 3.14
Bowel resection (%)	70.3
Partial/total colectomy (%)	51.4
Stoma (%)	
End ileostomy	29.7
Loop ileostomy	37.8
No stoma	2.7

**Table 5:** Specific Surgical Procedures in IC-CHD Group

Procedure	IC-CHD (N = 37)
Jejunostomy (%)	24.3
Ileostomy (%)	75.7
Colostomy (%)	8.1

**Table 6:** Mortality and Aristotle-Scores

Characteristic	CHD (N = 116)	IC-CHD (N = 37)	P-value
Mortality (%)	7.8	29.7	< 0.001 (C)
Aristotle Score (mean ± SD)	1.23 ± 2.25	7.46 ± 4.89	< 0.001 (M-W)

Cardiac diagnoses were stratified based on CHD complexity according to the ACC/AHA (American College of Cardiology/American Heart Association) Guidelines: mild, moderate and severe(Tables 2 and 3) [17, 18].

25.5% of all patients were born prematurely, with a mean gestational age of 36.6 weeks and a mean birth weight of 2790 g. A slight majority of 53.6% of all patients had non-cyanotic heart defects.

The mean age at the time of bowel surgery was 222 days, with a median of 41 days. The median time between cardiac and bowel surgery was 8 days. Bowel resection was necessary in 70.3% of the IC-CHD group, with 51.4% undergoing partial or total colectomy.

Gestational age *[33.6 weeks (SD:5.71) vs. 37.6 weeks (3.95), p* =  < *0.001]* and birth weight *[2040 g (1200) vs. 3080 g (867), p* =  < *0.001]* were significantly lower in patients with CHD who developed IC. Also mean Apgar-Scores after 1, 5 and 10 min each were significantly lower in the IC-CHD-group (Table 1). We did not see a significant difference in patients with cyanotic heart defects in between both groups *(p* = *0.269)*; the majority of both groups consisted of non-cyanotic heart defects *(IC-CHD* = *62.2% & CHD* = *50.9%)*. The complexity of heart defects did not differ significantly and was evenly distributed. In both groups. ECMO treatment was administered to 4 patients each (p = 0.097). Despite the mean ECMO duration for patients with IC being longer *(19.5 days vs. 2.5 days)*, this difference was not statistically significant. A significant difference was found in duration of CPB during heart surgery *[IC-CHD* = *40.8 min (78.3) vs. CHD* = *87.1 (61.1), p* =  < *0.001]* (Table 2).

Regarding the Aristotle-score, it was shown that the IC-CHD-Group had significantly higher scores than the CHD-group *[IC-CHD* = *7.46 (4.89) vs. CHD* = *1.23 (2.25), p* =  < *0.001]* (Table 6).

## Discussion

Ischemic colitis and congenital heart disease share the burden of being a major cause of infant morbidity and mortality. In this study, we aimed to identify risk factors for IC in pediatric patients with CHD. To our knowledge this is the first study to compare pediatric patients with CHD and surgically treated IC and those with CHD without surgically treated bowel ischemia.

The relationship between gestational age and the incidence of ischemic colitis in patients with congenital heart defects (CHD) is complex and multifaceted. Among term infants, CHD stands out as a crucial predisposing factor for IC. The risk of IC is notably higher in term infants with conditions such as hypoplastic left heart syndrome and truncus arteriosus [6]. However, studies have reported varying thresholds for increased risk. For instance, it was found that among very low birth weight (VLBW) neonates with serious CHD, the incidence of IC was highest at gestational ages below 32 weeks[12]. Another study reported an increased risk among premature infants with CHD when their birth weight or gestational age fell below thresholds of 30.5 weeks and 1250 g, respectively [19]. Further, the retrospective study reported an incidence rate of IC among premature infants with CHD as follows: 14.3% for those < 30 weeks gestation, 7.4% for those 30–31 weeks gestation, and 2.5% for those ≥ 32 weeks gestation. These stratified incidence rates underscore the critical role of gestational age in the development of IC. These thresholds are consistent with the findings of this study, where the IC-CHD group had significantly lower mean gestational age and birth weight. A further, multicenter study corroborates these findings with an incidence rate of IC among very low birth weight (VLBW, < 1500 g) neonates with serious CHD at 13%. This high incidence rate among VLBW neonates with CHD is consistent with the significantly lower mean birth weights observed in the IC-CHD group in the present study. Also, mortality was significantly higher in neonates with both CHD and IC (55%) compared to those with only CHD (34%) or only IC (28%). This study highlights that the presence of CHD increases the incidence and mortality of IC in VLBW neonates [12].

Only 16 patients in the IC-CHD group can be referred as term-born. In these 16 patients, the most common CHD was pulmonary atresia, followed by dextro-transposition of the great arteries (d-TGA). Both conditions result in the perfusion of the intestines with low-oxygenated blood [20, 21]. However, among neonates with CHD, the incidence of cyanotic heart defects did not significantly differ between those who developed IC and those who did not. This is supported by a case–control study with cardiac disease, which found that, after multivariable analysis, only prematurity and episodes of low cardiac output remained significantly associated with the development of NEC [11]. It can thus be hypothesized that, on the one hand, low tissue perfusion is a causative factor in the development of IC; if the oxygen partial pressure in the blood is also reduced, this can lead to an exacerbation of IC.

Additionally, hypoplastic Left Heart Syndrome (HLHS), Truncus Arteriosus and Aortopulmonary Window are associated with a higher risk of IC, likely due to reduced mesenteric perfusion mechanisms [11]. Infants with an Atrioventricular Canal Defect also exhibit a notably higher risk for developing IC compared to other congenital heart disease diagnoses [12]. It seems probable that over-shunting from left to right is the underlying cause, resulting in decreased perfusion of the abdominal organs, notably the intestines.

Multiple pregnancies were significantly more common in the IC-CHD group. This increased incidence could be attributed to the shared placental blood supply and other complications associated with multiple gestations, which can predispose these infants to NEC and other complications [22].

APGAR scores at 1, 5, and 10 min were significantly lower in the IC-CHD group, indicating poorer immediate post-birth health. Lower APGAR scores are often associated with higher risks of perinatal complications, including IC, particularly in infants with CHD [23].

ECMO use was more frequent and notably longer in the IC-CHD group, suggesting a more severe clinical course. On the other hand, the significantly shorter duration of cardiopulmonary bypass (CPB) in the IC-CHD group suggests that these patients might have undergone quicker, possibly less complex cardiac interventions due to their critical condition. The need for rapid intervention could reflect the urgency and severity of their clinical status [24]. It is also plausible that these patients underwent cardiac catheterization instead of surgery due to their limited condition. Furthermore, as discussed below, the IC-CHD group comprised patients with PDA as their underlying cardiac diagnosis who did not receive any CPB.

Bowel resection was necessary in 70.3% of the IC-CHD group, with 51.4% undergoing partial or total colectomy. These high rates of bowel resection highlight the severe gastrointestinal involvement in IC [25, 26]. Various stoma procedures were performed, with end ileostomies and loop ileostomies being the most common. The variability in stoma procedures underscores the need for individualized surgical approaches based on the extent and location of bowel necrosis.

The mean age at the time of bowel surgery was 222 days, with a median of 41 days, indicating a wide range in the timing of surgical intervention. The median time between cardiac and bowel surgery was 9 days, emphasizing the temporal relationship between these procedures. The time from the onset of IC to bowel surgery was short, with a median of 0 days, reflecting the urgency of surgical intervention once IC was diagnosed.

The lower gestational age and birth weight observed in the IC-CHD group not only contribute to the risk of IC but also complicate the surgical outcomes. Premature infants with CHD are at higher risk of perioperative complications, including prolonged hospital stays, increased need for postoperative support such as ECMO, and higher mortality rates [27]. These findings underscore the importance of gestational age and birth weight in predicting surgical outcomes and the need for specialized care for these high-risk infants.

The Aristotle score is a highly regarded tool for predicting postoperative outcomes in pediatric cardiac surgery, where higher scores reflect more complex and higher-risk procedures. Our study revealed that the IC-CHD group had significantly higher Aristotle scores (mean: 7.46 vs. 1.23, p < 0.001) with a mortality rate in the IC-CHD group (29.7% vs. 7.8%, p < 0.001), indicating more severe and complex heart defects. However, the data (as detailed in Table 5) demonstrate otherwise; the elevated scores were primarily attributed to baseline characteristics, such as prematurity or gestational weight, and not as such as the complexity of the heart defect itself. This is evidenced by the shorter CPB times observed.

Prematurity is a well-established risk factor for gastrointestinal complications, including necrotizing enterocolitis. In our cohort, the significant correlation between high Aristotle scores and poor postoperative outcomes, such as increased mortality, appears to be more closely linked to prematurity rather than CHD itself. This highlights the importance of adopting comprehensive risk assessment strategies that account for individual patient characteristics in pediatric cardiac care [26]. However, there is no doubt that premature babies with a complex CHD have the highest risk for developing NEC/IC.

This study offers a detailed analysis of risk factors and surgical outcomes in pediatric patients with congenital heart defects who develop ischemic colitis. The current collective may represent a combination of classical NEC, cardiogenic NEC and cases of postoperative and post-interventional ischemic colitis. Key risk factors identified include lower gestational age, reduced birth weight, multiple pregnancies, and poorer initial health status, as evidenced by lower APGAR scores. Additionally, higher Aristotle scores highlight the complexity and severity of these cases. In light of the evidence indicating the absence of significant clustering of IC, both in the complexity of the heart defect and in the incidence of cyanotic heart defects, it can be postulated that the development of IC even in patients with CHD is largely influenced by non-cardiac factors.

The surgical outcomes reveal the extensive and diverse interventions required to manage IC, underscoring the importance of individualized treatment approaches tailored to each patient’s unique condition. The significantly higher mortality rate observed in the IC-CHD group highlights the grave impact of IC in these patients and underscores the critical need for vigilant monitoring and timely intervention to improve outcomes.

The study has several limitations. The inclusion of patients with an isolated PDA was handled differently in the available studies. In our study, patients with PDA who underwent bowel surgery were allocated to the IC-CHD group. The PDA patients in our study were all extremely or very preterm infants with ELBW or VLBW. Therefore, it can be argued that these patients fall into the classic NEC rather than cardiogenic IC category [23, 26, 28–30]. However, the control group comprised six patients with a PDA, five of whom had ELBW, thereby facilitating a more accurate comparison.

Premature infants and those with congenital heart disease present distinct baseline characteristics such as gestational age, birth weight, and overall health status, which can independently influence the incidence and severity of ischemic colitis. These intrinsic differences complicate direct comparisons between the two groups. The underlying mechanisms of NEC and IC may differ; in premature infants, NEC is often linked to intestinal immaturity and dysbiosis, whereas in infants with CHD, IC may stem from reduced intestinal blood flow due to cardiac anomalies.

The risk factors for colitis also vary significantly between these populations. Premature infants are more likely to experience prolonged parenteral nutrition and antibiotic exposure, while infants with CHD are exposed to risks associated with surgical interventions and hemodynamic instability. Consequently, the required medical and surgical interventions differ; premature infants need specific nutritional support and care, whereas CHD infants require cardiac-related treatments that impact IC development and outcomes. This variation leads to different mortality and morbidity rates, further complicating direct comparisons [30, 31].

Comparative studies must meticulously adjust for confounding factors to ensure differences are not mistakenly attributed to baseline disparities rather than NEC itself. Ethical considerations are paramount in research involving vulnerable populations like premature infants and those with CHD, necessitating careful study design and data comparison to maintain ethical integrity. Despite these challenges, the study is a crucial addition to the scientific literature, enhancing our understanding of NEC and IC in these distinct yet overlapping populations and guiding more tailored clinical interventions.

## Data Availability

No datasets were generated or analysed during the current study.
